# Hormonal Contraceptive Use and the Prevalence of Endometriotic Lesions at Different Regions within the Peritoneal Cavity

**DOI:** 10.1155/2014/590950

**Published:** 2014-01-20

**Authors:** Brett D. McKinnon, Dominic Bertschi, Juilette Wanner, Nick A. Bersinger, Michael D. Mueller

**Affiliations:** Department of Obstetrics and Gynecology, Inselspital, Berne University Hospital, University of Berne, Effingerstrasse 102, 3010 Berne, Switzerland

## Abstract

Endometriosis is an estrogen-dependent disease that can lead to chronic pain and subfertility. Endometriotic lesions found in different locations are heterogeneous and may represent a collection of related but distinct conditions. Whether there is a relationship between hormonal contraceptive (HC) use and endometriosis is still controversial. The purpose of this study was to determine whether HC use affected the prevalence of endometriotic lesions differently based on lesion location. Data was retrospectively collected from 161 patients presenting to the Berne University Women's Hospital between 2008 and 2012 for laparoscopic investigation. Women with histologically proven endometriosis were included in the study and patients were grouped according to lesion location and HC use. The results of the study indicate that HC users are significantly less likely to have endometriotic lesions on the ovaries, although in contrast, no difference was observed in the incidence of lesions in the rectovaginal septum (RVS) or peritoneal region. In addition, women using HC who were diagnosed with endometriotic lesions on the peritoneum were significantly younger than women with lesions in other locations. In conclusion, women with endometriosis who are currently using HC are less likely to have ovarian endometriotic lesions than in alternate locations.

## 1. Introduction

Endometriosis is an estrogen-dependent disease defined by the presence, growth, and invasion of endometrial epithelial and stromal cells outside the uterine cavity that can lead to chronic pelvic pain and a reduced fecundity. It is an extremely prevalent condition with up to 10% of reproductive age women suffering from the disease [1] with an even higher incidence amongst infertile women [2] and adolescent girls with chronic pelvic pain [3]. Current first line treatment involves the induction of a hypoestrogenic state through the use of hormonal contraceptives (HC) or gonadotropin releasing hormone agonists (GnRHa). If unsuccessful the endometriotic lesions must be removed surgically.

Although the exact aetiology of endometriosis is not yet clear, certain factors such as lifestyle, nutrition, genetic polymorphisms, and environmental pollutants [4–6] are known to increase the potential for lesions development. Given the estrogen dependence of the disease HC use has also been investigated. Currently, however, the link between HC use and endometriosis is still debated as conflicting reports have shown both a reduced [7, 8], and increased risk [9, 10] as well as no association at all [11–13].

It is possible that the effects of HC use in endometriosis development are not yet clear because the inherent heterogeneity of endometriotic lesions that develop at different regions has not yet been sufficiently accounted for in these studies. Ectopic endometrial lesions can develop in multiple locations throughout the body with some examples even recorded in the lungs [14] and brain [15]. Predominantly, however, endometriotic lesions are found within the peritoneal cavity and can be broadly separated into three main regions: the rectovaginal septum (RVS), the peritoneum, and the ovaries. Biochemical and histological data show significant differences between lesions that develop at different locations leading to suggestions that lesions from these locations may have distinct pathophysiologies and should be classed as separate entities [16]. Moreover, lesions can occur in either one or more of these regions, and it is not yet clear whether multiple lesions are established separately, or their growth is facilitated by the accompanying inflammatory reaction.

Endometriosis is a disease of reproductive age women and a significant portion of this population use HC for both fertility control and the treatment of pathological conditions. The heterogeneity of endometriotic lesions suggests endometriosis could be considered a collection of related, but independent diseases, and thus, a thorough understanding of the effects of hormonal treatments and whether they are dependent on the specific presentation of the endometriotic lesion is vital. We have therefore performed a retrospective analysis of data collected from women presenting to our clinic to determine the prevalence of endometriotic lesions and whether this is significantly affected by HC use.

## 2. Materials and Methods

### 2.1. Patient Data

A cross sectional study was performed with data collected retrospectively from women presenting to the Bern University Women's Hospital during 2008–2012 for laparoscopic investigation of chronic pelvic pain or idiopathic infertility. Institutional ethics review board approval was obtained and women were included in the study if they provided informed consent and had a lesion removed during surgery that was histologically confirmed as endometriosis. All surgery was performed during the proliferative phase and the patient was staged according to the revised American Fertility Society Score (rAFS) [17]. The lesion locations were noted by the surgeon during the operation and were subsequently grouped into one of three possible regions; the RVS, ovarian, or peritoneum, as described previously [18]. Medical records were searched for relevant data.

During the study period we received informed consent from a total of 359 women, 213 of which had tissue excised during surgery and at least one lesion subsequently confirmed as endometriosis via histological examination. A further 52 women reported GnRHa use prior to surgery and were excluded from the study, resulting in a total of 161 women. The age range of women included in the study was 19–51 years with a mean of 32.56 ± 0.46 (Mean ± SEM).

To determine the prevalence of endometriotic lesions at the different locations, both with and without HC use, two separate groupings of women were made. The initial analysis was performed by dividing all women into one of two groups based on the presence or absence of a lesion in the defined region. Therefore, if a woman had a lesion on the ovaries, irrespective of whether additional lesions were identified at other locations, they were grouped as ovarian. If no lesion was present on the ovary the women were grouped as non-ovarian. This was repeated for both the peritoneal and RVS regions.

Grouping the women based on the exact region or combination of regions that endometriotic lesions were found allowed further analyses with more stringently defined groups. Based on this criteria, seven groups in total were possible, three of which represent single locations: (i) ovarian, (ii) RVS, or (iii) peritoneal, and four groups that represented combined regions: (i) peritoneal and ovarian, (ii) peritoneal and RVS, (iii) ovarian and RVS, or (iv) all three locations.

Hormonal treatment was determined by the self-reporting of women prior to surgery. HC use was split into either current or non-use. Current use was defined as use prior to and within the three months leading up to surgery, and non-use was defined as no use for a minimum of three months prior to surgery. Contraceptive use included all treatments used to prevent birth including oral (combined and gestagen only) and vaginal progesterone.

### 2.2. Statistical Analysis

Mean age was compared between two groups with a Student's *t*-test and between three groups with a one-way analysis of variance (ANOVA) test with a *post-hoc* Bonferroni's multiple comparison between groups. A Fisher's exact test was used to compare the number of women grouped by the presence or absence of endometriotic lesions within each region and the comparison between single and multiple lesions. A Chi-squared test was used to analyse the difference in prevalence between the three distinct regions (peritoneal, RVS, and ovarian). All statistical tests were performed with GraphPad Prism 5 for Mac OSX and the results were considered significant if the *P* value was below 0.05.

## 3. Results

### 3.1. The Incidence of Endometriotic Lesions at Different Locations and the Age of Patients

In 81 of the 161 women (81/161; 50.3%) an endometriotic lesion was found in only one of the three defined regions, while 80 patients (80/161; 49.7%) had endometriotic lesions in more than one (multiple) of the defined regions. The most common single region was the peritoneum with lesions found in 37 (37/161; 23.0%) women. The RVS (22/161; 13.7%) and ovarian (22/161; 13.7%) region had a similar incidence with 22 each. The most common combination of multiple regions was the peritoneum and ovaries with 42 (42/161; 26.1%) occurrences, followed by a combination of all three regions with 21 (21/161; 13.0%) cases. A combination of lesions in the peritoneum and RVS was found in 14 (14/161; 8.7%) women and the combination of RVS and ovarian lesions was uncommon with only 3 (3/161; 1.9%) cases observed in the total study population (Table 1).

Of the 161 women included in this study 88 (88/161, 54.7%) were non-HC users, whereas 73 (73/161, 45.3%) were HC users. The mean age of the HC users (30.66 ± 0.66) was significantly younger (*P* = 0.0001) than women who were non-HC users (34.14 ± 0.60). When ages were compared between the absence vs. presence groups there was a significant difference (*P* = 0.0003) in the peritoneal group with HC users (29.49 ± 0.79, *n* = 49) younger than non-HC users (33.43 ± 0.70, *n* = 61). A similar difference was also observed in the ovarian group, as HC users (31.41 ± 1.15, *n* = 32) were significantly younger (*P* = 0.0218) than non-HC users (34.09 ± 0.72, *n* = 57). In the single lesion groups, the women with peritoneal lesions that were HC users (27.91 ± 1.20, *n* = 23) were significantly younger (*P* = 0.0114) compared to non-HC users (33.36 ± 1.71, *n* = 14) (Table 1).

When comparing ages across lesion locations there was no significant difference (*P* = 0.0971) between the ages of women based on presence of a lesion at the defined region. If the samples were split by HC and non-HC use, again, there was no significant difference between the age of the women based on the presence of a lesion. When only women with a lesion in a single location were taken into account a one-way ANOVA test confirmed a significant difference between the mean ages (*P* = 0.0058). A *post-hoc* Bonferroni's Multiple comparison test indicated that women with peritoneal lesions were significantly younger (29.97 ± 1.07, *n* = 37) than women with lesions in either the RVS (34.40 ± 1.20, *n* = 22; *P* < 0.05) or the ovaries (34.73 ± 1.30, *n* = 22; *P* > 0.05). When these women were further split based on HC use a one-way ANOVA test indicated a significant difference existed between the HC users (*P* = 0.0434) but not in the non-HC users (*P* = 0.4932) (Table 1).

### 3.2. Hormonal Contraceptive Use and the Region Specific Prevalence of Endometriotic Lesions

Of the entire study population, 54.7% (88/161) of the women were currently non-HC users compared to 45.3% (73/161) who were current HC users. Of the 81 women with endometriotic lesions in a single region, 41 (41/81; 50.6%) were non-HC users, whereas 40 (40/81; 49.4%) were current HC users. Of the 80 women with endometriotic lesions in multiple regions 47 (47/80; 58.8%) were non-HC users compared to 33 (33/80; 41.3%) who were current HC users. The difference between the prevalence of lesions in either single or multiple locations based on current HC use was not significant (*P* = 0.3435) (Table 2).

When grouped via the simple presence or absence of endometriotic lesions the most common location was the peritoneal region with 110 cases (110/161; 68.3%). Ovarian lesions were detected in 89 cases (89/161; 55.3%) and RVS lesions in 58 (58/161; 36.0%) cases. In the study group, there was a significantly lower probability (OR = 0.45, 95% CI 0.24–0.88, *P* = 0.0167) of having an endometriotic lesion present (32/89; 36.0%) on the ovary compared to it being clear of a lesion (40/72; 55.6%) if the women were current HC users. In contrast, no significant effect of current HC use was observed on the incidence of endometriotic lesions in either the peritoneum or the RVS (Table 2).

To confirm an association between the reduced prevalence of ovarian endometriotic lesions and HC use we selected women with lesions in a single region only and used a Chi-squared test to analyse the distribution amongst these locations between current HC users and non-HC users. In these women, the incidence of ovarian endometriotic lesions, compared to the peritoneum and the RVS, was also significantly lower (*P* = 0.0347) with current HC use (Table 3).

## 4. Conclusion

The relationship between HC use and endometriosis is still controversial. We proposed that the heterogeneity of endometriotic lesions that are found in different regions might contribute to the disparity in results previously reported. This study reports that current HC users are less likely to have endometriotic lesions on the ovaries, whereas in contrast, no difference was observed in the incidence of lesions in either the RVS or peritoneal regions. The results therefore suggest that HC use may affect endometriotic lesions at separate locations differently and may be indicative of distinct pathophysiologies for lesions that develop at separate locations. Although this study only represents a small sample set it also suggests a potentially protective effect of HC use on the development of ovarian endometriotic lesion, which should be investigated further.

Previous evidence indicates that significant biochemical and anatomical differences exist between lesions at different locations and that it is possible that lesions found in the peritoneal cavity, the ovaries, and in the RVS all have a distinct pathophysiologies [16]. HC use has been suggested to influence the incidence of endometriosis [19]; however whether it affects all lesions equally has not been assessed and thus may offer further evidence for distinct pathophysiologies based on the location of the lesions or underlying tissue. This study was therefore designed to assess the relationship between endometriosis and HC use while accounting for the heterogeneity that may exist between endometriotic lesions that develop in separate regions. A particular strength of this study is that we have identified the location of every lesion removed from the patients and as such have been able to perform a dual analysis with two distinct patient groupings. Firstly, an analysis was performed based simply on the presence or absence of a lesion at the defined location. Secondly, a smaller but more stringent grouping was made that included only women with lesions in a single region. By using the larger cohort of women based simply on the presence or absence of a lesion, we had larger sample numbers in each group and therefore more statistical power. The results of this analysis show it was significantly less likely for women who have endometriosis and are using HC to have a lesion on the ovaries than either the peritoneal cavity or the RVS. Subsequently, by studying women with lesions confined to a single region only we have analysed a smaller but more distinct subgroup that may help to eliminate potential confounding factors introduced when women have more than one affected region. Most importantly, the results of this analysis confirmed those of the larger cohort.

Previously, only a limited number of studies on HC use and endometriosis have taken into account the location of the lesions, or when they have done so this aspect was not a primary focus as a potential source of heterogeneity. These studies have also used different methods of grouping patients and as such have also reported disparate results. One study by Chapron et al. (2011) examined both the location of the lesion as well as the primary reason for HC prescription. This study found that past, but not current HC use, was frequently associated with the development of deep infiltrating endometriosis (DIE) [20], particularly when prescribed for severe primary dysmenorrhea, but found no relationship with HC use and endometriomas. Contrary to our study, this report grouped women based on the most serious lesion identified, rather than their presence or absence. Superficial peritoneal endometriosis was considered the least severe, followed by endometrioma and finally DIE as the most severe. Therefore, based on these groupings, a number of women with endometriomas may have also been included in the DIE group, or women with peritoneal lesions may have been included in both the endometrioma and DIE group. This represents a significant difference from the groupings performed in our study and could explain the variation in results. Another study that focused primarily on environmental risk factors found a lower but nonsignificant previous use of HC with women who had either peritoneal or DIE. This study did not report on ovarian endometriosis [21].

It must also be noted that in this study we have only analysed women with histologically confirmed endometriosis. We have not analysed the incidence of endometriosis in the population, but rather the incidence of endometriosis at defined regions in a population of women with endometriosis. By performing the analysis in this way we have eliminated a non-endometriosis group that is notoriously difficult to establish as a homogenous sample and have focused on the incidence of endometriosis at different locations in a group of women known to be susceptible to endometriosis. Whether HC use has a similar effect on women that are not originally susceptible to endometriosis is not addressed in this study.

As with all studies some inherent weakness and limitation are also present and thus should be discussed. Firstly, the mean age of the current HC users was significantly younger than those who were non-HC users. A further analysis of the mean ages shows that women with lesions in both the peritoneal cavity and the ovaries, but not the RVS, are also significantly younger in the current HC users group compared to the non-HC users, although in the single lesion group this difference exists only in the peritoneal samples and not the ovarian or RVS samples. This difference between the age of the current HC users and non-HC users is not surprising as the primary reason for HC use (contraception) will be more common amongst younger women. These demographic differences in the use of HC will therefore always make this difficult to resolve in such studies.

This difference in age between the current HC users and non-HC users also raises the possibility that the lower incidence of ovarian endometriotic lesions observed in women using HC may be due to their younger age. Previous studies have found that the incidence of endometriosis increases with increasing age, with one study showing a peak between 30 and 35 [7] and another showing a peak between 40 and 44 [22], although both suggest that a difference of at least 5 years is required to achieve a significant difference. While there is a statistically significant difference between the ages of the two groups in our study, the entire difference is less then 5 years which is unlikely to be sufficient to alter incidence of the disease, particularly as a delay in the diagnosis from the original inception of the lesion can be up to 11 years [23]. In addition, although the age difference was statistically significant with both the peritoneal and ovarian groups we only observed a difference in the incidence of lesions based on current HC use in the ovarian group and not the peritoneal group. In fact, the incidence of ovarian endometriosis in current HC users was still much lower than that observed in peritoneal lesions even though the mean age of women with peritoneal lesions was significantly lower than that of women with ovarian lesions. The role of age in the incidence of endometriotic lesions at different locations should be taken into account when designing further studies to analyse the effect of HC use on lesions from different locations.

Previous studies have also suggested that the influence of HC use on the incidence of endometriotic lesions depends on whether the usage is still current. Women currently taking HC have a reduced incidence of endometriosis, whereas women who had stopped taking HC for over 12 months have an increased incidence [7, 22]. Past users were also shown to have an increased incidence of DIE, but not other types of endometriosis [20]. A meta-analysis of 18 studies also concluded that there was an increased incidence in past users, but a decreased incidence in current users [19]. As previous studies have shown that the duration of HC use is not related to disease status [7], but rather whether or not use is current, we focused only on current use and whether it influences the incidence of endometriotic lesions at different locations. We used a three-month period to establish current use, as this is a period commonly needed for a stable menstrual cycle to be reestablished after hormonal control. Although we used this three-month cut-off we do not suggest that abstinence from HC for three months is sufficient to allow the growth of endometriosis. It is most likely that non-HC users have not taken HC for a period much longer than this. Unfortunately, we did not have a large enough sample size to analyse the impact of the duration of use.

Multiple theories have been proposed to explain the aetiology of endometriosis [24] and although retrograde menstruation is the most widely accepted it cannot explain the occurrence of all lesions, particularly those that occur in the absence of a functioning endometrium [25, 26], supporting the possibility of multiple aetiologies. It is possible that the results of this study are a reflection of the inherent differences that exist in lesions that develop at different anatomical locations and that these lesions may have variable aetiologies potentially influenced by HC use. This is not surprising given the variability of the underlying tissue where endometriotic lesions can be found.

The results of this study therefore showed that endometriotic lesions are less likely to be found on the ovaries than in other locations in current HC users. These results suggest a potential protective effect of HC use that differs based on the anatomical locations most susceptible to the disease. It is important to note that the results do not suggest that HC use protects against endometriosis, but rather only that it may affect development at separate locations differently. It must be remembered, however, that no causality can be drawn from this study, and further work should be performed to understand exactly what the link is between HC use and the development of location specific endometriotic lesions.

## Figures and Tables

**Table 1:** The incidence and mean age of patients with endometriotic lesions at defined locations in both the current HC use or non-HC use groups.

	*n* (%)	Age			*P*
		All	No HC	HC	
All women	161		88	73	
Age		32.56 ± 0.46	34.14 ± 0.60	30.66 ± 0.66	0.0001^1^
Absence versus presence					
Peritoneal	110	31.67 ± 0.55 (*n* = 110)	33.43 ± 0.70 (*n* = 61)	29.49 ± 0.79 (*n* = 49)	0.0003^1^
RVS	58	33.28 ± 0.71 (*n* = 58)	34.30 ± 0.93 (*n* = 30)	32.18 ± 1.105 (*n* = 28)	0.1340^1^
Ovarian	89	33.12 ± 0.56 (*n* = 89)	34.09 ± 0.72 (*n* = 57)	31.41 ± 1.15 (*n* = 32)	0.0218^1^
*P* value		0.0971^2^	0.7033^2^	0.0771^2^	
Single region	81/161 (50.3)	32.47 ± 0.85 (*n* = 81)			
Peritoneal	37/161 (23.0)	29.97 ± 1.07 (*n* = 37)	33.36 ± 1.71 (*n* = 14)	27.91 ± 1.20 (*n* = 23)	0.0114^1^
RVS	22/161 (13.7)	34.4 ± 1.2 (*n* = 22)	36.09 ± 1.55 (*n* = 11)	32.73 ± 1.81 (*n* = 11)	0.1736^1^
Ovarian	22/161 (13.7)	34.73 ± 1.3 (*n* = 22)	35.50 ± 1.64 (*n* = 16)	32.67 ± 2.03 (*n* = 6)	0.3507^1^
Single location (*P* value)		0.0058^2^	0.4932^2^	0.0434^2^	
Multiple regions	80/161 (49.7)	32.65 ± 0.58 (*n* = 80)			
Peritoneal and ovarian	42/161 (26.1)	32.88 ± 0.80 (*n* = 42)	33.96 ± 0.99 (*n* = 27)	30.93 ± 1.22 (*n* = 15)	0.0680^1^
Peritoneal and RVS	14/161 (8.7)	32.57 ± 1.67 (*n* = 14)	32.83 ± 3.14 (*n* = 6)	32.38 ± 1.95 (*n* = 8)	0.8985^1^
RVS and ovarian	3/161 (1.9)	33.67 ± 4.41 (*n* = 3)	—	33.67 ± 4.41 (*n* = 3)	—
Peritoneal, ovarian, & RVS	21/161 (13.0)	32.10 ± 0.94 (*n* = 21)	32.71 ± 1.14 (*n* = 14)	30.86 ± 1.70 (*n* = 7)	0.3668^1^
Multiple location (*P* value)		0.9333^2^	0.7389^2^	0.7873^2^	
Single versus multiple (*P* value)		0.8462^1^			

^1^Comparison of the mean age between HC users and non-HC users, as well as between the single and the multiple regions, was performed by a Student's *t*-test. ^2^Comparison of the mean age between women with lesions in different locations, as well as for both the single and multiple regions, was performed by a one-way ANOVA test with a *post-hoc* Bonferroni's multiple comparison test.

**Table 2:** Hormonal contraceptives and the presence of endometriotic lesions in defined regions.

	Single region, *n* (%)	Multiple region, *n* (%)	OR (95% CI)	*P* value
Single versus multiple regions				
All	81	80		
No hormone	41 (50.6)	47 (58.8)		
HC	40 (49.4)	33 (41.3)	0.72 (0.39–1.34)	0.3435

	Absence, *n* (%)	Presence, *n* (%)	OR (95% CI)	*P* value

Ovarian				
All	72	89		
No hormone	32 (44.4)	57 (64.0)		
HC	40 (55.6)	32 (36.0)	0.45 (0.24–0.88)	0.0167
Peritoneal				
All	51	110		
No hormone	28 (54.9)	61 (55.4)		
HC	23 (45.1)	49 (44.5)	0.98 (0.50–1.91)	1.000
RVS				
All	101	58		
No hormone	57 (56.4)	30 (51.7)		
HC	44 (43.6)	28 (48.3)	1.21 (0.63–2.31)	0.6210

A Fisher's exact test was used to determine if the incidence of endometriotic lesions in particular locations was significantly different under HC use.

**Table 3:** Prevalence of women with endometriotic lesions in single regions only with and without hormonal contraceptive use.

	Ovarian, *n* (%)	Peritoneal, *n* (%)	RVS, *n* (%)	*P* value
Single location cases				
No hormone *n* (%)	16 (39.0)	14 (34.1)	11 (26.8)	0.0347
HC *n* (%)	6 (15.0)	23 (57.5)	11 (27.5)	

A Chi-squared test was used to determine whether the incidence of endometriotic lesions in different locations was significantly different under HC use.
